# Transcriptome-based analysis of the effects of compound microbial agents on gene expression in wheat roots and leaves under salt stress

**DOI:** 10.3389/fpls.2023.1109077

**Published:** 2023-05-10

**Authors:** Chao Ji, Zengwen Liang, Hui Cao, Zhizhang Chen, Xuehua Kong, Zhiwen Xin, Mingchao He, Jie Wang, Zichao Wei, Jiahao Xing, Chunyu Li, Yingxiang Zhang, Hua Zhang, Fujin Sun, Jianlin Li, Kun Li

**Affiliations:** ^1^ College of Seed and Facility Agricultural Engineering, Weifang University, Weifang, Shandong, China; ^2^ Key Laboratory of Biochemistry and Molecular Biology in University of Shandong Province, Weifang University, Weifang, Shandong, China; ^3^ Taishan Forest Ecosystem Research Station, Key Laboratory of State Forestry Administration for Silviculture of the Lower Yellow River, Shandong Agricultural University, Taian, Shandong, China; ^4^ Shandong Yongsheng Agricultural Development Co., Ltd., Yongsheng (Shouguang) Vegetable Technology Research Institute Co., Ltd, Weifang, China; ^5^ College of Foreign Languages, Weifang University, Weifang, Shandong, China; ^6^ Weifang Hanting Vestibule School, Weifang Education Bureau, Weifang, Shandong, China; ^7^ Runxin Fruit and Vegetable Cultivation Cooperative of Weifang Economic Development Zone, Weifang Agricultural Bureau, Weifang, Shandong, China; ^8^ Weifang Nuode Biotechnology Co., LTD, Weifang Agricultural Bureau, Weifang, Shandong, China; ^9^ College of Forestry, Shandong Agriculture University, Taian, Shandong, China

**Keywords:** transcriptomic analysis, salt stress, plant growth-promoting rhizobacteria, wheat, gene expression

## Abstract

**Introduction:**

Salt stress inhibits the beneficial effects of most plant growth-promoting rhizobacteria. The synergistic relationship between beneficial rhizosphere microorganisms and plants helps achieve more stable growth-promoting effects. This study aimed 1) to elucidate changes in gene expression profiles in the roots and leaves of wheat after inoculation with compound microbial agents and 2) to determine the mechanisms by which plant growth-promoting rhizobacteria mediate plant responses to microorganisms.

**Methods:**

Following inoculation with compound bacteria, transcriptome characteristics of gene expression profiles of wheat, roots, and leaves at the flowering stage were investigated using Illumina high-throughput sequencing technology. Gene ontology (GO) function and Kyoto Encyclopedia of Genes and Genomes (KEGG) enrichment analyses were performed on the genes that were significantly differentially expressed.

**Results:**

The expression of 231 genes in the roots of bacterial preparations (BIO) -inoculated wheat changed significantly (including 35 upregulated and 196 downregulated genes) compared with that of non-inoculated wheat. The expression of 16,321 genes in leaves changed significantly, including 9651 upregulated genes and 6670 downregulated genes. The differentially expressed genes were involved in the metabolism of carbohydrates, amino acids, and secondary compounds as well as signal transduction pathways. The ethylene receptor 1 gene in wheat leaves was significantly downregulated, and genes related to ethylene-responsive transcription factor were significantly upregulated. GO enrichment analysis showed that metabolic and cellular processes were the main functions affected in the roots and leaves. The main molecular functions altered were binding and catalytic activities, among which the cellular oxidant detoxification enrichment rate was highly expressed in the roots. The expression of peroxisome size regulation was the highest in the leaves. KEGG enrichment analysis showed that linoleic acid metabolism expression was highest in the roots, and the expression of photosynthesis-antenna proteins was the highest in leaves. After inoculation with a complex biosynthesis agent, the phenylalanine ammonia lyase (PAL) gene of the phenylpropanoid biosynthesis pathway was upregulated in wheat leaf cells while 4CL, CCR, and CYP73A were downregulated. Additionally, *CYP98A* and *REF1* genes involved in the flavonoid biosynthesis pathway were upregulated, while F5H, HCT, CCR, E2.1.1.104, and TOGT1-related genes were downregulated.

**Discussion:**

Differentially expressed genes may play key roles in improving salt tolerance in wheat. Compound microbial inoculants promoted the growth of wheat under salt stress and improved disease resistance by regulating the expression of metabolism-related genes in wheat roots and leaves and activating immune pathway-related genes.

## 1 Introduction

Yellow River Delta is an important reserve granary in China. Soil salinization is an important factor restricting wheat production in this region. A large number of studies have confirmed the importance of plant growth-promoting rhizobacteria (PGPR) in achieving higher crop yield and improving crop quality and soil fertility (Primieri et al., 2021; Liu et al., 2022). However, most of the beneficial rhizobacterial strains were poor colonizers of saline-alkali soil, and their effect on wheat growth enhancement was not stable. This restricts the role of microbial agents in agricultural production in saline-alkali land (Coban et al., 2022). With a view to reduce harmful fertilizer use while maintaining food security, the creation of safe and efficient microbial agents has become a major demand. Halotolerant PGPRs with 1-aminocyclopropane-1-carboxylate deaminase (ACCD) activity can reduce ethylene accumulation, prevent oxidative stress, and promote plant growth under salt stress, which has become an important microbial resource to promote the growth of crops in saline-alkali land (Etesami and Beattie, 2018).

Compared with a single strain, this compound microflora had the advantages of higher environmental adaptability and biological activity. For example, Zhang et al. (2022) detected the core flora in maize stems by high-throughput sequencing technology and isolated *Klebsiella variicola* MNAZ1050 and *Citrobacter* sp. MNAZ1397 with the entire nitrogen-fixing gene cluster (*nif*). Applying the compound microbial agent prepared by these two strains and the other two auxiliary strains to the farmland can increase the nitrogen accumulation of maize by 11.8%. The compound microbial agent composed of *Arthrobacter* sp. AT5 and *Halobacillus* sp. NY15 also showed higher biological activity and could degrade atrazine more efficiently (Xu et al., 2019). Therefore, it is beneficial to promote the sustainable development of agriculture in saline-alkali land by screening and applying functional bacteria with high biological activity and stable colonization to form compound microbial agents. Studying the plant response mechanism mediated by these microbial agents might help us further investigate and improve such compound microbial agents.

*Bacillus velezensis* JC-K3 and *Bacillus subtilis* HG-15 are two strains of rhizosphere-beneficial bacteria with both growth-promoting and disease-preventing functions (Ji et al., 2021; Ji et al., 2022b). The two strains have no antagonistic effects and can stably colonize wheat roots in saline-alkali land. Both strains have the characteristics of producing ACCD, indole-3-acetic acid (IAA), siderophore, and proline. They have clear effects in promoting wheat growth and can systematically induce wheat to synthesize more soluble sugars and proteins, thereby reducing salt stress damage and promoting plant root development and nutrient absorption (Ji et al., 2021; Ji et al., 2022a). In particular, inoculation with JC-K3 increases the abundance of bacterial communities in branches and leaves, reduces the abundance of fungal communities, and reduces the richness and diversity of bacterial and fungal communities in wheat roots.

In recent years, RNA sequencing technology has been widely used in abiotic and biotic stress response research in plants. Many salt-stress response-related genes involved in different metabolic processes have been identified, enriching the information on plant stress resistance regulatory networks (Zhang et al., 2020). Amirbakhtiar et al. (2019) analyzed root transcriptome sequences from an Iranian salt-tolerant wheat variety (Arg) and proposed that genes related to transporters, phenylpropanoid biosynthesis, transcription factors, glycosyltransferases, glutathione metabolism, and plant hormone signal transduction play an important role in wheat response to high-salt stress. Currently, studies on the mechanism of action and colonization of halotolerant PGPR strains have mostly focused on the function of a single strain and plant physiological indexes and rhizosphere microflora response, while only a few studies investigated plant root and leaf gene expression in response to compound microbial agents (Ausubel, 2005; Jones and Dangl, 2006). In this study, transcriptome sequencing technology was used to study the changes in gene expression profiles of wheat roots and leaves under salt stress after inoculation with *Bacillus velezensis* JC-K3 and *Bacillus subtilis* HG-15 to further understand the mechanisms of PGPR from the perspective of plants.

## 2 Materials and methods

### 2.1 Biocontrol strain and culture medium

The microbial inoculant was a compound agent of *Bacillus subtilis* HG-15 and *Bacillus velezensis* JC-K3. Luria–Bertani (LB) liquid medium was used as the seed and fermentation medium, according to the method described by Ji et al. (2020). When spore formation in the fermentation liquid was > 95%, diatomite (sterilized at a high temperature, 121 °C, 20 min) was added at a proportion of 10% to the fermented liquid. The bacteria were allowed to adsorb onto the diatomite, and the suspension was centrifuged at 3,100 × *g* for 20 min. The supernatant was removed, and the sediment was stored at −40°C for 48 h before being placed in a lyophilizer (Labconco FreeZone^®^ Plus 4.5 L; Kansas City, MO, USA), and treated at −48°C and 9 Pa for 48 h. The HG-15 and JC-K3 densities in the resultant solid microbial agents were 472 × 10^8^ CFU g^−1^ and 511 × 10^8^ CFU g^−1^, respectively. The aforementioned bacterial preparations were mixed with sterile diatomite and diluted to 20 × 10^8^ CFU g^−1^.

### 2.2 Experimental design

An experimental plot system was established in the Weifang Changyi area of Shandong Province (WF), China (119°31′55″E, 36°38′47″N) between October 2021 and July 2022. The initial chemical properties of the soil were as follows: pH, 8.11; electrical conductance, 316 μs cm^-1^; organic matter, 23.51 g kg^-1^; total nitrogen, 1.792 g kg^-1^; available nitrogen, 79.35 mg kg^-1^; Olsen-P, 18.83 mg kg^-1^; and exchangeable potassium, 97.06 mg kg^-1^. The abundances of culturable bacteria and fungi in the soil were 5.73×10^4^ CFU g-1 dry weight of soil and 2.44×10^3^ CFU g^-1^ dry weight of soil, respectively. This study used a completely randomized block design with three replicates per treatment. The treatments were BIO and non-inoculated wheat (CK). Each replicate consisted of an area of 40 m^2^ (8 m × 5 m) isolated by a buffer zone.

The wheat variety *Jimai* 22, which was mainly cultivated in the Yellow River Delta area, was used as the test wheat variety. Seeds were surface-sterilized with 1% sodium hypochlorite for 5 min, washed 3–5 times with sterile water, and sown on the plot at 0.6 kg per 40 m^2^ on October 12, 2021. At the jointing stage, the wheat seedlings in the treatment groups were irrigated with bacterial preparations (dissolved in water at 5.0 kg per 40 m^2^) on February 20, 2022, and again on March 08, 2022; the wheat seedlings in the control group were irrigated with the same volume of tap water. Basal fertilizer (45% Yangfeng compound fertilizer, N14-P16-K15; 1.2 kg per 40 m^2^) and nitrogen fertilizer (urea, 1.5 kg per 40 m^2^) were applied prior to planting and during the green-up period, respectively.

### 2.3 RNA extraction, quality detection, and sequencing library construction from wheat roots and leaves

The root and leaf tissues of wheat at the flowering stage were cut, wrapped, labeled with a tin foil paper, and quickly placed in a liquid nitrogen tank for freezing. The total RNA of the CK and BIO samples was extracted using an RNAprep Plant Kit (Tiangen, China). Based on a previous study (Meng et al., 2019), the mRNA was first purified and then fragmented for cDNA synthesis. The synthesized cDNA was subjected to end repair and then 3’-adenylation. The adapters were ligated to the 3’-adenylate ends of the cDNA fragments. After the PCR reaction, the product was purified and dissolved in ethidium bromide (EB) solution. The library was verified on an Agilent Technologies 2100 biological analyzer and amplified to prepare DNA nanospheres (DNBs). The DNB was loaded into a patterned nanoarray and produced a single-ended 150-base reading in a combined probe-anchored synthesis (cPAS) manner.

### 2.4 Sequencing data analysis and differential expression gene screening

The sequencing data were uploaded to the NCBI SRA database (https://www.ncbi.nlm.nih.gov/sra/SRA, Accession No.: PRJNA905537) and aligned with the reference genome using Hisat2 software (CsaV3_1, http://www.cucurbitgenomics.org/organism/20) (Kim et al., 2015). Trimmomatic software was used to preprocess the original data, and the reads in the whole quality control process were statistically summarized (Bolger et al., 2014). Genes with |_log2_FC|>1 and *P* < 0.05 were considered differentially expressed genes (DEGs) (Love et al., 2014).

### 2.5 Gene ontology and Kyoto encyclopedia of genes and genomes enrichment analysis of differentially expressed genes

Gene Ontology (GO) function analysis was performed using the Blast2GO and topGOR packages to obtain functional modules for all significant differential genes, i.e., upregulated and downregulated differential gene enrichment. Pathway analysis of differentially expressed protein-coding genes was performed using the Kyoto Encyclopedia of Genes and Genomes (KEGG) database (combined with KEGG annotation results), and the significance of differential gene enrichment in each pathway entry was calculated using the hypergeometric distribution test (Conesa et al., 2005; Minoru et al., 2008).

### 2.6 qRT−PCR analysis

Total RNA was extracted, and the cDNA was synthesized as previously described (Meng et al., 2019). *TaACTIN* (GenBank: AB181991) was used as the endogenous control. A PerfectStart Green qPCR SuperMix (TransGen Biotech, Beijing, China) kit was used for quantitative real-time polymerase chain reaction (qRT-PCR). The 20-µl reaction mixtures contained 1 µl template, 0.4 of each primer (10 µM), 10 µl SuperMix (Beijing Quanshijin Biotechnology Co., Ltd., China), and 8.2 µl ddH_2_O. The amplification protocol was 94°C for 30 s, followed by 40 cycles of 94°C for 5 s, 60°C for 30 s, and 72°C for 30 s. All cDNA and qRT-PCRs were carried out in triplicate. The 2^-△△Ct^ method was used to calculate the relative expression of each gene (Pfaffl, 2001). Three biological replicates were used in the experiments.

### 2.7 Statistical analyses

Excel 2017 software and GraphPad Prism 7.0 software were used to analyze the data. Sequence data (raw data or raw reads) and raw image data from the Illumina HiSeq 2500 sequencing were converted into a FASTQ format file by base calling (Cock et al., 2009). The sequence data contained the reads and quality of the bases, and high-quality clean reads for subsequent analysis were obtained after removing and filtering raw reads containing adapters, excessive N, or low-quality bases. The transcriptome data alignment software TopHat2 was used to align all clean reads with the reference genome sequence of common wheat (Kim et al., 2013). Gene expression was calculated using aligned reads to ensure high accuracy. The calculated expression was measured using the fragments per kilobase of transcript per million mapped reads (FPKM) (Mortazavi et al., 2008). Differentially expressed genes were screened using the DESeq2 software (|_log2_FC| ≥ 1 and adjusted p-value [padjust] < 0.05).

## 3 Results

### 3.1 Transcriptome sequencing, assembly, and differentially expressed genes analysis

Transcriptome analysis of 20 samples was completed, and a total of 225.65 Gb of clean data was obtained. The clean data of each sample was more than 10.3 Gb in size, and the percentage of Q30 bases was greater than 92.33%. The GC content was approximately 45% (**Table 1**). The Reference gene source was *Triticum aestivum*, the reference genome version was IWGSC, and the reference genome source was http://plants.ensembl.org/Triticum_aestivum/Info/Index. The clean reads of each sample were aligned with the designated reference genome, and the alignment rate ranged from 73.35% to 93.67%. A total of 113669 expressed genes were detected, including 120588 known genes and 6919 new genes. A total of 185577 transcripts were found, including 146191 known transcripts and 39386 new transcripts.

**Table 1:** Data statistics of transcriptomics analysis.

Sample	Clean reads	Clean bases	Error rate (%)	Q20 (%)	Q30 (%)	GC content (%)
WF_CK_R1	80300466	11721250503	0.0263	97.42	93.14	54.73
WF_CK_R2	76745458	11174453419	0.0267	97.25	92.75	54.82
WF_CK_R3	73918364	10794331849	0.0272	97.07	92.34	54.27
WF_CK_R4	76910042	11141562859	0.0266	97.31	92.84	54.79
WF_CK_R5	72203552	10300074359	0.0269	97.24	92.62	54.62
WF_Bio_R1	75162678	10882740568	0.0271	97.09	92.40	54.37
WF_Bio_R2	75817238	10965706593	0.0268	97.22	92.63	54.22
WF_Bio_R3	76137288	11047211468	0.0268	97.23	92.67	54.50
WF_Bio_R4	75405302	10939520638	0.0270	97.16	92.53	54.62
WF_Bio_R5	78445538	11411466245	0.0270	97.14	92.46	54.16
WF_CK_L1	78897704	11564129927	0.0272	97.08	92.33	51.27
WF_CK_L2	83094062	12122551681	0.0271	97.12	92.41	49.83
WF_CK_L3	81685536	11986347986	0.0266	97.31	92.81	50.47
WF_CK_L4	79664264	11578299363	0.0269	97.18	92.60	52.28
WF_CK_L5	76927010	10944948800	0.0267	97.28	92.79	52.52
WF_Bio_L1	80299412	11869009647	0.0262	97.46	93.15	53.67
WF_Bio_L2	77608000	11265779607	0.0263	97.44	93.11	55.24
WF_Bio_L3	77945228	11388117173	0.0263	97.39	93.06	55.32
WF_Bio_L4	73883258	10811486103	0.0268	97.25	92.66	53.69
WF_Bio_L5	79789564	11742839159	0.0267	97.23	92.71	55.12

**Figure 1:**
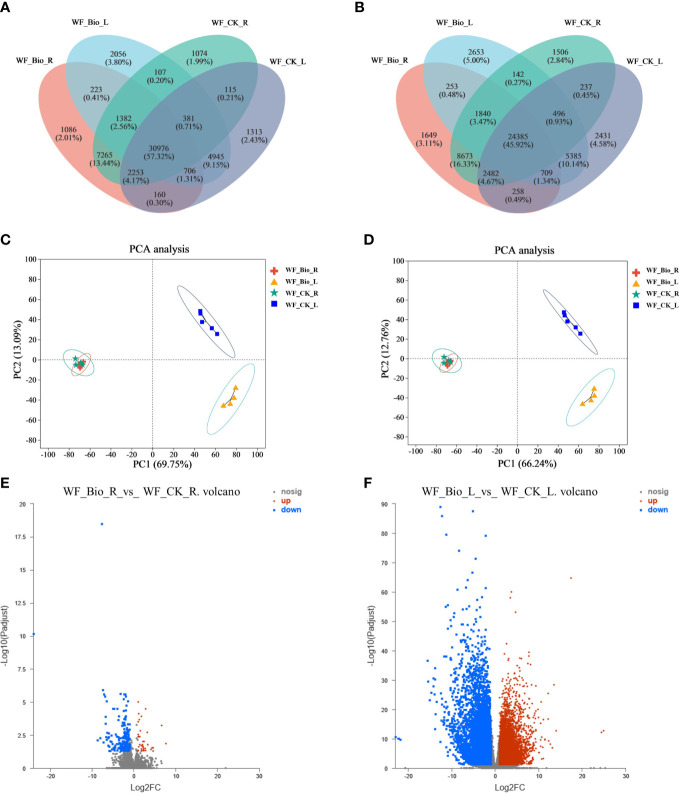
Venn **(A, B)**, PCA analysis **(C, D)** and expression difference analysis **(E, F)** of samples based on the expression matrix. Venn analysis showed co-expressed and uniquely expressed genes **(A)** and transcripts **(B)** between groups. Circles of different colors represent genes/transcripts screened based on expression levels in a group, and values represent the number of common and unique genes/transcripts between different groups. PCA analysis is based on the expression of the sample gene **(C)** and transcript **(D)** clustering. After dimensionality reduction analysis, there are relative coordinate points on the principal component. The distance of each sample point represents the distance of the sample. The closer the distance, the higher the similarity between the samples. The horizontal axis represents the contribution of principal component 1 (PC1) to distinguishing samples, and the vertical axis represents the contribution of principal component 2 (PC2) to distinguishing samples. Expression difference analysis identified differentially expressed genes. The ordinate is the statistical test value of the difference in gene expression, namely the p value. The abscissa is the fold change of gene expression between the two samples. Each point in the figure represents a specific gene. The red points represent a significantly up-regulated gene, the green points represent a significantly down-regulated gene, and the gray points show genes with no significant differential expression. **(E)** are the genes in roots; **(F)** are the genes in leaves.

Venn analysis can determine the number of co-expressed and uniquely expressed genes or transcripts between samples or groups. In this study, transcriptome sequencing of roots and leaves treated with CK and BIO was performed, and genes with tissue-specific expression in different treatments and common expression genes were obtained. GO enrichment analysis was performed on the genes specifically expressed in each tissue to understand their main biological functions. In all, 1086 genes and 1649 transcripts were specifically expressed in BIO-treated wheat roots. The number of genes and transcripts exceeding our threshold for expression CK-treated wheat roots were 1074 and 156, respectively. The number of genes and transcripts in BIO-treated wheat leaves were 2056 and 2653, respectively. The genes and transcripts that exceeded our criteria for significance in wheat leaves treated with CK were 1313 and 2431, respectively. In total, 30,976 genes and 34,385 transcripts were co-expressed in each treatment (**Figures 1A, B**; **Supplementary Tables 1, 2**). Furthermore, principal component analysis (PCA) was performed for both CK and BIO samples. The similarity between genes and transcripts in wheat root samples treated with CK and BIO was high (concentrated mainly in the third quadrant). The wheat leaves samples of CK group were concentrated in the second quadrants, and the BIO group was concentrated mainly in the fourth quadrants, indicating that there were significant differences and good repeatability between genes and transcripts in leaf samples (**Figures 1C, D**; **Supplementary Tables 3**, **4**). Comparative analysis of BIO and CK treatment at the transcriptional level was based on the expression of quantitative results and differential gene analysis between groups. The results showed 197 differentially expressed genes between the two groups in wheat roots, of which 51 were upregulated, and 146 were downregulated. There were 14619 differentially expressed genes in leaves, of which 8367 were upregulated, and 6252 were downregulated (**Figures 1E, F**; **Supplementary Table 5**).

Following inoculation with compound bacteria, 9 auxin response factor genes, including *TraesCS1D02G337400.1*, *TraesCS3A02G246000.1*, and *TraesCS3B02G486000.1* and 15 auxin-responsive protein-coding genes, including *TraesCS3D02G162700.1, TraesCS5A02G058600.1*, and *TraesCS5B02G386800.1* were significantly upregulated in wheat leaves (*P* < 0.05). However, there were no significant differences in the wheat roots. The *TraesCS2D02G314900.3* gene related to zeaxanthin epoxidase and chloroplastic was significantly upregulated in roots and leaves (*P* < 0.05). *TraesCS4A02G274300.1*, associated with ethylene receptor 1, was significantly downregulated in wheat leaves, whereas *TraesCS5B02G214400.1* and *TraesCS3B02G357600.1*, associated with ethylene-responsive transcription factor (ERF), were significantly upregulated. The expression levels of five bZIP-related genes were significantly different in wheat roots and leaves. *TraesCS3B02G411300*, *TraesCS3D02G371900*, *TraesCS7D02G171300*, and *TraesCS6D02G312800* were down-regulated in wheat leaves and up-regulated in roots, all of which were involved in the map04075 pathway (name: ABF, KO id: K14432, definition: Plant hormone signal transduction). The *TraesCS1A02G072600* gene was not involved in map04075 and was down-regulated in wheat roots and leaves (**Supplementary Tables 5**-**8**).

### 3.2 Functional annotation analysis

**Figure 2:**
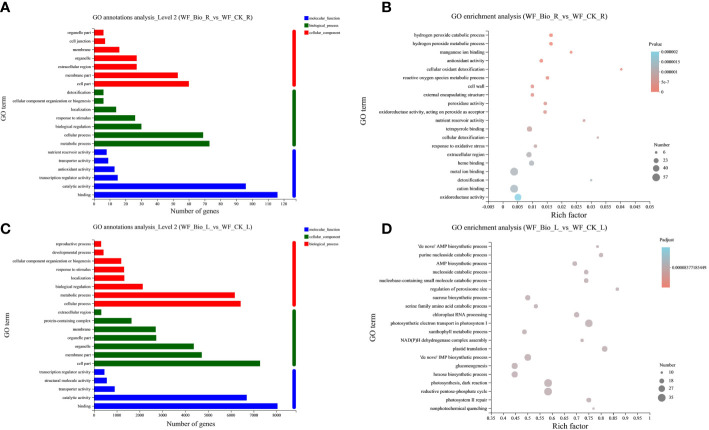
GO classification and enrichment statistics. The ordinate represents the GO term. The abscissa of **(A, C)** represents the number of genes and transcripts of the GO term, respectively. The abscissa of **(B, D)** represents Rich factor, that is, the ratio of the number of enriched genes **(B)**/transcripts **(D)** (Sample number) to the number of annotated genes/transcripts (Background number) in the GO term, respectively. The larger the Rich factor, the greater the degree of enrichment. The size of the point represents the number of genes/transcripts in this GO Term, and the color of thes point corresponds to different Padjust ranges. The GO term of the top 20 abundances is shown.

GO is a database established by the Gene Ontology Consortium that can classify genes in a selected gene set. The GO categories were developed to evaluate potential DEG functions. Among biological processes, roots (69, 73) and leaves (6171, 6425) have the most GO terms in metabolic and cellular processes. Among the molecular functions, roots (116, 96) and leaves (8040, 6691) have the most GO terms in binding and catalytic activity have the most genes. Among the cellular components, roots (60, 53) and leaves (7277, 4722) have the most GO terms in cell and membrane parts (**Figures 2A, C**). GO enrichment analysis was performed on the obtained differential genes to show the enrichment results of the top 20. The detoxification rate of cellular oxidants was the highest in roots. The regulation of peroxisome size had the highest enrichment in leaves. The results showed that metal ion binding, cation binding, and oxidoreductase activity were the main biological processes in roots. Photosynthetic electron transport in photosystem I, photosynthesis, dark reactions, and the reductive pentose-phosphate cycle were the main biological processes in leaves (**Figures 2B, D**).

**Figure 3:**
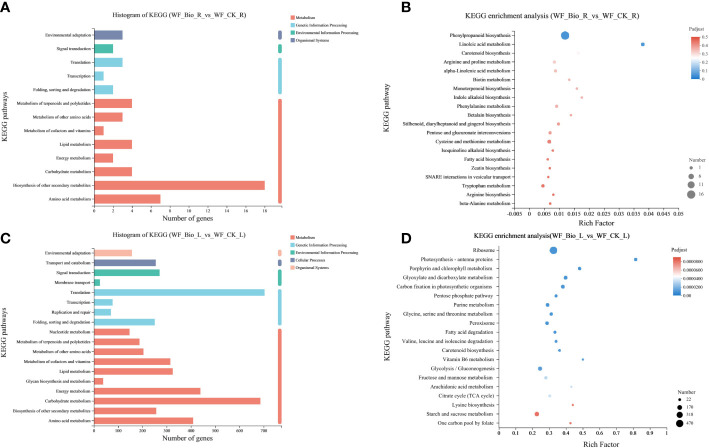
Kyoto Encyclopedia of Genes and Genomes (KEGG) enrichment analysis. The ordinate is the name of the KEGG metabolic pathway; the abscissa of **(A, C)** denotes the number of genes or transcripts released into this pathway, respectively. The abscissa of **(B, D)** represents Rich factor, that is, the ratio of the number of enriched genes **(B)**/transcripts **(D)** (Sample number) to the number of annotated genes/transcripts (Background number) in the KEGG pathway, respectively. The larger the Rich factor, the greater the degree of enrichment. The size of the point represents the number of genes/transcripts in this KEGG pathway, and the color of the point corresponds to different Padjust ranges. The KEGG pathway of the top 20 abundances is shown.

KEGG is a knowledge base for systematic gene function analysis and association of genomic and functional information. Using the KEGG database, the genes in the gene set were classified according to the pathways involved or functions performed. In wheat roots, more genes (18) in Metabolism were annotated to Biosynthesis of other secondary metabolites; more genes (3) in translation were annotated to Genetic Information Processing; more genes (2) in Environmental Information Processing were annotated to Signal Transduction; and more genes (3) in Organismal Systems were annotated to Environmental adaptation. In wheat leaves, more genes (686) in Metabolism were annotated to Carbohydrate metabolism; more genes (703) in Genetic Information Processing were annotated to Translation, more genes (271) in Environmental Information Processing were annotated to Signal transduction; more genes (255) in Cellular Processes were annotated to Transport and Catabolism; and more genes (157) in Organismal Systems were annotated to Environmental adaptation. KEGG pathway analysis showed that 4 and 686 genes were involved in carbohydrate metabolism in the roots and leaves, respectively. Seven and 409 genes were involved in Amino Acid Metabolism; a total of 18 and 257 genes were involved in pathways such as Biosynthesis of Other Secondary Metabolites (**Figures 3A, C**). The obtained differential genes were subjected to KEGG enrichment analysis to show the enrichment results of the top 20. The enrichment rate of linoleic acid metabolism was the highest in the roots. Photosynthesis-antenna proteins showed the highest enrichment rate in leaves. Phenylpropanoid biosynthesis was the main biological process in roots. Ribosome, starch and sucrose metabolism, and glycolysis/gluconeogenesis were the main types of biological processes in leaves (**Figures 3B, D**).

**Figure 4:**
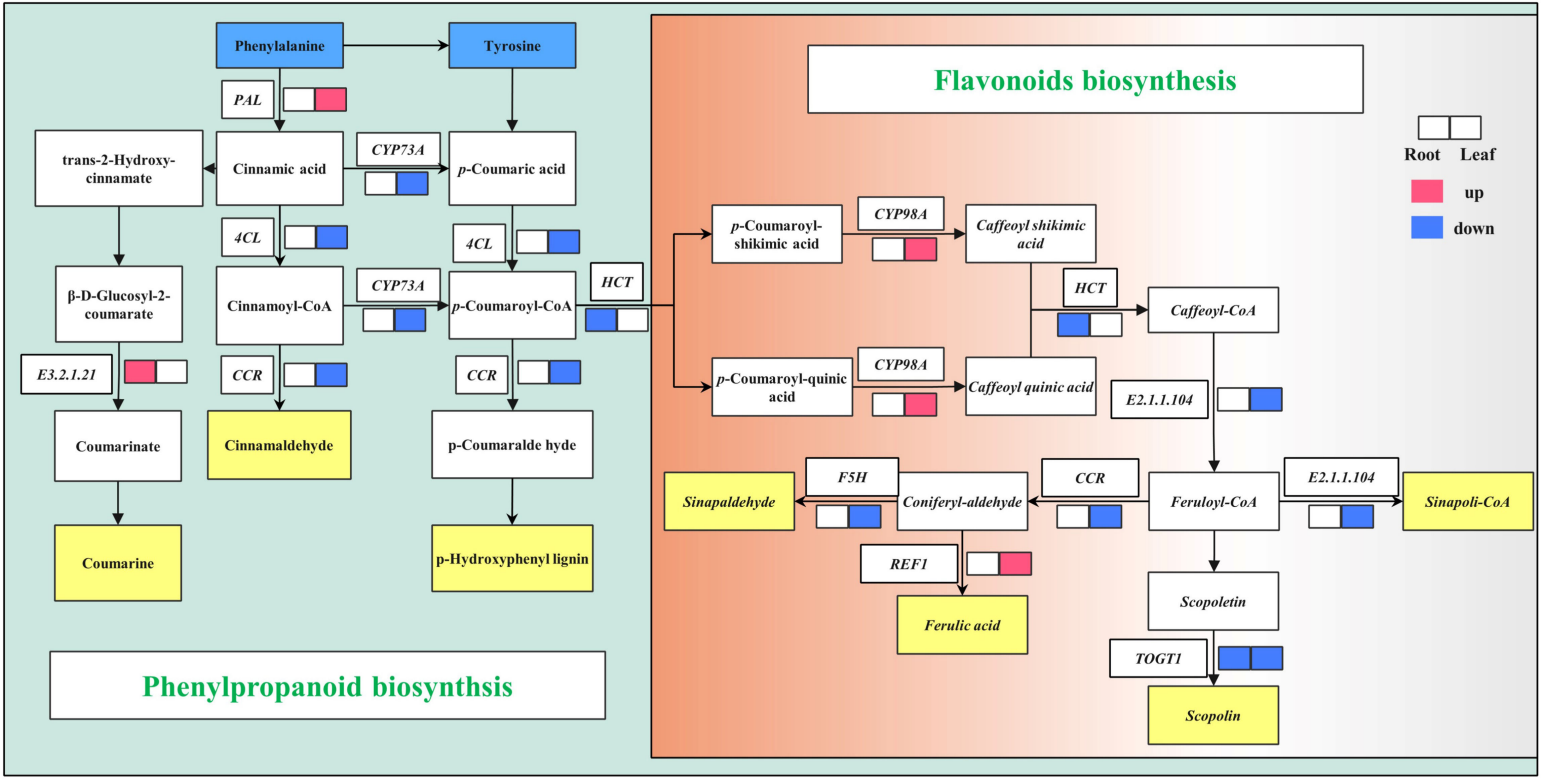
Phenylpropanoid and flavonoid biosynthesis pathway. Red and blue indicate upregulated and downregulated genes. *PAL*, phenylalanine ammonia-lyase; *CYP73A*, trans-cinnamate 4-monooxygenase; *4CL*, 4-coumarate-CoA ligase; *CCR*, cinnamoyl-CoA reductase; *HCT*, shikimate O-hydroxycinnamoyltransferase; *F5H* (*CYP98A*), ferulate-5-hydroxylase; *REF1*, coniferyl-aldehyde dehydrogenase; *TOGT1*, scopoletin glucosyltransferase; *E2.1.1.104*, caffeoyl-CoA O-methyltransferase; *E3.2.1.21*, beta-glucosidase.

The effect of adding compound bacteria on secondary metabolic pathways is shown in **Figure 4**. The gene *E3.2.1.21* involved in the phenylpropanoid biosynthesis pathway was upregulated, and the genes *HCT* and *TOGT1* involved in the flavonoid biosynthesis pathway were downregulated. The PAL gene involved in the phenylpropanoid biosynthesis pathway was upregulated, and *4CL*, *CCR*, and *CYP73A* were downregulated in wheat leaf cells after inoculation with compound bacteria. The genes *CYP98A* and *REF1*, involved in the flavonoid biosynthesis pathway, were upregulated, while *F5H*, *HCT*, *CCR*, *E2.1.1.104*, and *TOGT1* were downregulated (**Figure 4**).

**Figure 5:**
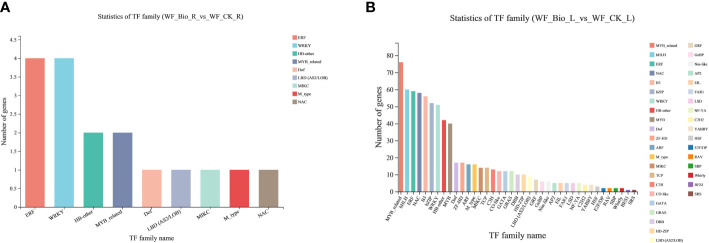
Transcription factor family statistics. The abscissa is different transcription factor families; the ordinate is the number of genes belonging to the transcription factor family. **(A)** are the TF family in roots; **(B)** are the TFs in leaves.

The differentially expressed genes (Hmmscan E-value: 0.00001) of wheat in the control (CK) and BIO were obtained by statistical analysis of the obtained transcription factor families. The results covered 47 transcription factors (TFs), and the gene and transcript numbers were 5893 and 9093, respectively. The expression of most TFs was affected by the application of compound bacteria. *ERF*, *WRKY*, *HB*-other, and *MYB*-related TFs were relatively large TFs in the roots. *MYB*-related, *bHLH*, *ERF*, *NAC*, and *B3* were relatively large TFs in leaves and are the main families known to be involved in stress tolerance mechanisms. The results showed that the expression of these TFs changed significantly in response to the compound microbial agents and significantly influenced the mechanism of microbial agents under salt stress (**Figure 5**).

**Figure 6:**
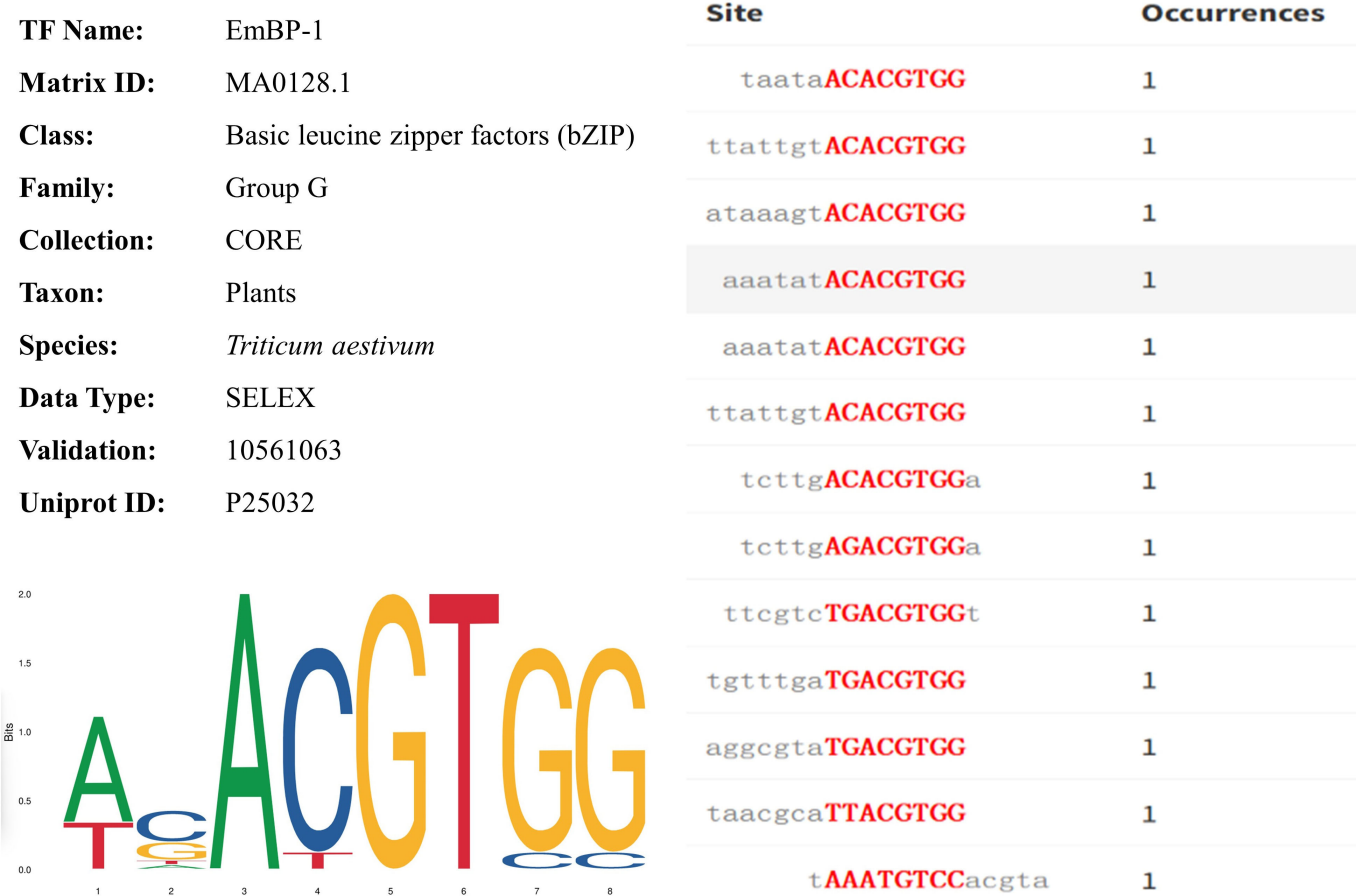
The profile summary, sequence identification and binding site sequence of MA0128.1.

Using the plantTFDB (*Triticum aestivum*) database to compare the key DEGs between BIO and CK, many DEGs were identified as transcription factors. The 22 transcription factors in roots mainly belonged to MIKC, ERF, and WRKY (**Supplementary Table 9**). The 1409 transcription factors in leaves mainly belonged to bHLH, MYB-related, bZIP, and B3 (**Table 1**). A total of 19 transcription factors were obtained by comparing the gene with the JASPAR database (*Triticum aestivum*) information. After removing redundant transcription factors, TraesCS5D02G473000, TraesCS5B02G470600, TraesCS2D02G236200, TraesCS5A02G460800, TraesCS2A02G245700, MSTRG.78111, and TraesCS2B02G269600 genes were identified, which were distributed on chromosomes 5D, 5B, 2D, 5A, 2A, 7D, and 2B, respectively (**Table 1**). The DNA binding domains annotated by these seven genes were PF00170 (bZIP transcription factor, https://www.ebi.ac.uk/interpro/). The target genes of these seven genes were predicted, and 21261 information was obtained, including 5125 genes (**Table 1**). All genes corresponded to MA0128.1 (TF id), EmBP-1 (Motif id, transcription factor binding site id in the database) (**Table 1**). Among them, in wheat leaves, TraesCS5B02G470600 (Location: 643978490-643981969, length: 3480) was significantly down-regulated compared with uninoculated compound bacteria. **Figure 6** shows the information, sequence identification, and binding site sequence of MA0128.1.

### 3.3 Validation of RNA sequencing results by qRT-PCR

To verify the reliability of the transcriptomic data, we screened eight differentially expressed genes for qRT-PCR verification. The expression trends measured by the qRT-PCR assay validated the RNA sequencing results, which indicated a high degree of reproducibility between transcript abundances assayed using RNA-Seq and the expression profiles revealed by qRT-PCR data (**Figure S1** and **Supplementary Table 1, 4**).

## 4 Discussion

Determining plant salt-stress response mechanisms will provide valuable information for improving crop salt tolerance by PGPR. Significant progress in understanding plant salt-stress responses has been achieved recently through molecular genetics and genomics analysis. However, there is a lack of research using transcriptomics to investigate gene expression in wheat under different cultivation and management measures or to investigate the effects of halotolerant PGPRs on gene expression. The compound bacteria used in this study were two strains of growth-promoting bacteria that can enhance the growth of wheat under salt stress and prevent and control pathogen infection. Changes in the gene expression profile of Jimai 22 under salt stress treatment with and without a compound microbial agent were analyzed by RNAseq. After the wheat plant was inoculated with the compound agent, the expression of cellular oxidant detoxification was highest in the roots, while the expression of regulatory genes controlling peroxisome size was highest in the leaves. Metal ion binding, cation binding, and oxidoreductase activity were the main biological processes in roots (**Figure 2B**). Photosynthetic electron transport in photosystem I, photosynthesis, dark reactions, and the reductive pentose-phosphate cycle were the main types of biological processes in leaves (**Figure 2D**). This indicated that the BIO treatment mobilized the primary and secondary metabolisms and finally regulated the expression of related genes through signal transduction and ion transport, which clearly induced the salt stress defense responses and had the greatest effects on ion absorption and antioxidant activity of wheat root cells and on the photosynthesis of leaf cells (Abdelgawad et al., 2022; Ji et al., 2022a).

Unsaturated fatty acids in plants are synthesized by fatty acid desaturase, which plays an important role in plant growth and abiotic stress (Moradi et al., 2022). Linoleic acid (LA) is a polyunsaturated fatty acid essential for eukaryotes development (Chodok et al., 2013). In plants, a higher LA content is conducive to maintaining the integrity and fluidity of the cell membrane, which is beneficial for plant adaptation to environmental stresses (Dar et al., 2017; Miao et al., 2019). Qian et al. (2023) found that, LA metabolism may alleviate the membrane damage caused by high saline–alkali stress. The products and pathways of LA metabolism play an important role in the response of rice to high saline-alkali stress. In this study, the enrichment rate of LA metabolism was the highest in the roots (**Figure 3**). The results indicated that compound microbial agents may affect the function of membranes by changing the structure and fluidity of membranes, thereby improving the salt tolerance of wheat.

Salt stress can stimulate plants to produce excessive ethylene. Elevated ethylene levels in nodules reduce nitrogen fixation, thereby inhibiting further growth of plant roots and plants themselves. Reducing stress-induced ethylene levels reduces crop stress damage. PGPR, which secretes ACCD, reduces ethylene levels by metabolizing ACC, the precursor of ethylene production in plants. It can also affect endogenous ethylene homeostasis and promote root elongation by altering the genes encoding ethylene synthase, ACC synthase, and ACC oxidase (Ma et al., 2002; Etesami and Beattie, 2018). Ethylene-responsive transcription factors enhance plant resistance by controlling osmotic regulators. For example, *VaERF3* in *Vigna angularis* and *OsERF71* in rice can induce proline accumulation under saline-alkali stress and drought stress, respectively, to alleviate the corresponding stress resistance (Li et al., 2018; Li et al., 2019). In this study, both strains produced ACCD, and the transcriptome results showed that the ethylene receptor 1-related gene *TraesCS4A02G274300.1* was significantly downregulated and the ERF-related genes *TraesCS5B02G214400.1* and *TraesCS3B02G357600.1* were significantly upregulated in the wheat leaves inoculated with the compound microbial inoculants (**Supplemental Table 7**). This indicated that the wheat leaves inoculated with the compound microbial agent regulated ethylene accumulation and initiated the ethylene-based stress defense system. Moreover, ERF can also regulate abscisic acid (ABA) signaling from the source by regulating ABA synthesis-related genes, such as the ABA synthesis-related gene *NtSDR*, to increase ABA content and enhance the resistance of tobacco to salt and low-temperature stress (Wu et al., 2007).

IAA signal transduction pathway is closely related to plant growth and stress resistance (Steffen et al., 2008). The concentration of IAA in crops decreased significantly after salt stress (Dunlap and Binzel, 1996). For example, salt-tolerant strains *B*. *enodophyticus*, *B*. *equilensis*, and *Planococcus*, which were isolated from the rhizosphere of Salicornia europaea, were re-inoculated to *S*. *uropaea*; the germination rate and stem length increased by 7–11% and 13–22%, respectively. Root length increased by 44–57% and fresh weight increased by 21–54% (Zhao et al., 2013). A large number of studies have shown that inoculation with auxin producing PGPR can regulate IAA production in halophytes and sweet soil plants (Wang et al., 2009; Dodd et al., 2010). In this study, the regulation of auxin-responsive protein genes by compound microbial agents may be an important factor in enhancing salt tolerance in wheat (**Supplementary Table 7**).

Zeaxanthin epoxidase can catalyze the epoxidation reaction from zeaxanthin to violaxanthin and is a key enzyme in the lutein cycle, which can protect the plant photosynthetic apparatus during environmental stress. It can be used as an antioxidant in the plasma membrane to quench reactive oxygen species to reduce oxidative damage (Wang et al., 2010). In this study, genes related to zeaxanthin epoxidase and chloroplastic genes were significantly up-regulated in wheat roots and leaves that were inoculated with complex bacteria, indicating that the strain could maintain normal physiological activity by regulating the synthesis of zeaxanthin and chlorophyll in wheat (**Supplementary Tables 6**, **7**).

To adapt to a changing environment, plants must constantly adjust the distribution of energy and metabolites to maintain vigorous growth during different developmental processes and survive under stress. Metabolic flux reorientation can occur between primary and secondary metabolism, phenylpropanoid metabolism, and other secondary metabolic pathways, between different branches of the phenylpropanoid metabolic pathway. To maintain the dynamic balance of phenylpropane metabolism, the vigorous growth of plants should be maintained, and plants should resist biotic and abiotic stresses (Dong and Lin, 2020). The main biosynthetic pathways of secondary metabolites include the phenylpropanoid and the flavonoid biosynthesis pathways, which mainly involve genes that play important roles in plant growth, development, and responses to stress, including avoiding stress damage and improving plant disease resistance (Colquhoun et al., 2011; Xu et al., 2013). MYB, one of the largest transcription factor families in plants, is involved in regulating the phenylpropanoid metabolic pathway. *AtMYB20* enhances salt tolerance by inhibiting *PP2C* expression in *Arabidopsis thaliana* (Cui et al., 2013). This indicates that MYB transcription factors can participate in the ABA signaling pathway. MYB acts as a positive and negative regulator to actively participate in the response of plants to salt stress.

Flavonoid biosynthesis is derived from the phenylpropanoid metabolic pathway and is induced by Carbon metabolism (C state). The C state is considered a key factor in accumulating secondary metabolites (Liu et al., 2010). In this study, *Cinnamaldehyde, p-Hydroxyphenyl lignin*, *Scopolin*, *Sinapaldehyde*, and *Sinapoli-CoA* were downregulated in leaves (**Figure 4**), while photosynthesis-antenna proteins had the highest expression levels (**Figure 3**). This may be because the application of compound microbial agents reduced the stress on wheat and enhanced photosynthesis in the leaves and the intensity of the phenylpropanoid metabolic response. Wheat without compound microbial agents still needed to regulate gene expression to deal with salt stress and maintain the dynamic balance of phenylpropanoid metabolism.

The PAL pathway plays an important role in host defense against pathogens. A metabolomic study revealed that the resistance of the resistant wheat variety Sumai-3 to *Fusarium* head blight (FHB) was mainly due to the association of phenylpropanoids with flavonoid metabolites (Gunnaiah and Kushalappa, 2014). Consistently, many genes in the phenylpropanoid and flavonoid metabolic pathways were differentially expressed in the interaction between wheat and *Fusarium graminearum*. For example, comparative transcriptomic studies have found that the expression level of the PAL pathway key gene *CCoMT* in resistant wheat is much higher than that in the susceptible mutant Meh0106 (Ding et al., 2011). Transcriptomic analysis of the FHB-resistant durum wheat mutant showed that nearly one-third of the affected 181 secondary metabolic genes were PAL pathway genes (Kumar et al., 2020). Similarly, a transcriptome study of a winter wheat cultivar (Centenaire) showed that the PAL pathway gene *C4H* was upregulated after inoculation with *F. graminearum* (Muhovski et al., 2012). Transcriptomic analysis of four wheat cultivars, Nyubai, Wuhan1, HC374, and Shaw, showed that aromatic amino acid metabolism-related genes, particularly tryptophan biosynthesis-related genes, were upregulated after inoculation with *F. graminearum*. Tryptophan is the precursor of many phenylpropanoids and lignins, which help strengthen the primary cell wall and prevent fungal expansion in plant tissues (Walter et al., 2009). In this study, the PAL pathway in leaves was upregulated (**Figure 4**), indicating that the compound microbial agent activated the defense response of wheat, which may be an important mechanism by which the compound microbial agent reduced the incidence of wheat diseases.

Transcription factors can be induced under stress conditions, and signal transduction can reduce stress damage to plants in many ways. They play important roles in plant growth and development. The main transcription factor families that respond to salt stress include WRKY, MYB, NAC, AP2/ERF, ERF, Bhlh/HD*-*ZIP, bZIP, and C2H2. Transcriptome analysis of cotton under salt stress showed that the expression of WRKY and bHLH genes was upregulated, while the C2H2 gene was downregulated. Many transcription factors recognizing AP2 were involved in the stress response, and MYB and NAC genes were also highly upregulated (Cai et al., 2014). In this study, the expression of most TFs was affected by the application of compound bacteria. ERF, WRKY, HB-other, and MYB-related are major TF families in roots. MYB-related, bHLH, ERF, NAC, and B3 are major TF families in leaves and are the main TF families known to be involved in stress tolerance mechanisms (**Figure 5**).

WRKY transcription factors are mainly involved in biological processes such as crop senescence, seed development, germination, and dormancy and regulate the adaptability of plants to biotic and abiotic stresses. They often show different expression patterns under salt stress and can affect the expression of proline, sugars, and some antioxidants (Jiang and Deyholos, 2006). *GmWRKY16* enhances drought and salt tolerance in *Arabidopsis via* an ABA-mediated pathway (Ma et al., 2019). However, the expression of *TcWRKY53* is downregulated in *Thlaspi arvense* under salt stress (Wei et al., 2008). This indicates that the mechanisms of *WRKY13* and *WRK53* in the transcription factor family differ in response to salt stress. *WRKY13* acts as a positive regulator, while *WRKY53* acts as a negative regulator to regulate salt stress. In terms of regulating plant immunity and the stress response, HG-15 strains produce hormones such as salicylic acid and jasmonic acid (JA), thereby stimulating the host’s induced systemic resistance and improving its resistance to subsequent pathogen inoculation. Transcriptome sequencing in this study revealed that the expression levels of WRKY and ERF coding genes in wheat inoculated with compound bacteria were higher than those in the control. ERF transcription factors belong to the AP2/ERF family. In addition to osmotic adjustment substances and ethylene accumulation, *AtERF1* is also involved in ethylene- and jasmonate-mediated defense signaling pathways and can induce the expression of downstream defense-related genes (Lorenzo, 2002). This indicates that compound microbial inoculants could induce plant immunity and improve the disease resistance of the host by activating the expression of immune pathway genes in wheat roots and leaves.

The NAC transcription factor family exists only in plants and is involved in plant growth and development, fruit ripening, and hormone regulation. It is also involved in a series of response processes of plant stress (Shao et al., 2015). bZIP is mainly involved in the ABA pathway, which can bind to ABRE cis-acting elements to regulate downstream genes in response to various plant stresses (Sarna et al., 2008). The *AtbZIP2* gene, a member of the bZIP family in *Arabidopsis thaliana*, is downregulated under high salt stress and is involved in regulating SnRK-like kinases. The expression of *AtbZIP11* is upregulated in leaves and does not change in roots (Weltmeier et al., 2009). This indicates that the bZIP family not only responds to salt stress under sucrose induction but also participates in other signal transduction pathways, and different bZIP family members have different regulatory mechanisms (Liu et al., 2014; Zhang et al., 2017). In this study, after the application of compound microbial agents, the expression levels of five bZIP-related genes were significantly down-regulated in leaves (**Supplementary Tables 7**, **8**). We speculate that this may be related to the fact that the BIO-treated wheat leaves are less salt-stressed than the CK group and therefore did not stimulate the up-regulation of these genes.

The interrelationship among the TFs was complex. Further investigations focusing on the functions of these microbial-plant interactions TFs would greatly help decipher the molecular regulatory mechanisms of wheat response to microorganisms in the field. The upstream TFs would be worth consideration to be verified experimentally before further usage. TFs control gene expression by directly binding to a cis-regulatory element, the TF binding site (Motif) in the target gene promoter. Therefore, Motif provides valuable information for revealing the abundance and temporal/spatial patterns of gene expression (Lu et al., 2019). Interestingly, we found that the transcription factors TraesCS5D02G473000, TraesCS5B02G470600, TraesCS2D02G236200, TraesCS5A02G460800, TraesCS2A02G245700, MSTRG.78111, and TraesCS2B02G269600 correspond to EmBP-1 (**Table 1**), indicating that EmBP-1 regulates the corresponding target genes in a flexible manner during wheat-PGPR interaction. Consistent with previous studies, the identification of EmBP-1 is of great significance for understanding and constructing transcriptional regulation models of plant biological processes (Kuang et al., 2021).

Plants respond to salt stress through a series of complex metabolic networks and quickly predict the related factors of plant response to salt stress, which can reveal the defense mechanisms of plant responses to salt stress, such as signal transduction and energy metabolism. Transcriptomics plays an important role in studying plant defense mechanisms, including those in response to salt stress, and in breeding salt-tolerant varieties. The mechanism of plant response to salt stress is still poorly understood, and although some key genes have been discovered, there is still a long way to go to study the defense regulation mechanisms of the plant salt stress response.

## 5 Conclusion

In this study, Transcriptome-based analysis was used to study the changes in gene expression profiles of wheat roots and leaves under salt stress after inoculation with JC-K3 and HG-15 to further understand the mechanisms of PGPR from the perspective of plants. Our findings confirmed that inoculation with salt-tolerant bacteria significantly influences wheat gene expression. The effects of the compound microbial agents were prominent in leaves rather than roots. The primary reason for the improvement of disease resistance among wheat plants is attributed to the upregulation of genes in the PAL pathway following the application of compound bacteria. The key mechanism by which the compound microbial agents improve wheat stress tolerance may involve the upregulation of WRKY- and ERF-associated genes. This study provides novel insights into the ability of salt-tolerant microorganisms to improve salt tolerance and disease resistance in wheat; however, a single transcriptomic study cannot fully explain the mechanism by which microorganisms improve salt tolerance in wheat, hence further investigations remain warranted. Future studies should also include plant proteomics, metabolomics, and genetics to systematically study the defense mechanisms of plants in response to salt stress.

## Data availability statement

The datasets presented in this study can be found in online repositories. The names of the repository/repositories and accession number(s) can be found in the article/**Supplementary Material**.

## Author contributions

CJ, ZC, and XK conceived the ideas and designed the experiment. CJ, KL, ZC, XK, and ZX collected and analyzed the samples. CJ, KL, FS, CL, JL, YZ, HZ, and JX analyzed the data. CJ, ZC, KL, MH, ZL, and HC prepared the figures and wrote the manuscript. CJ, KL, ZL, and HC revised and edited the draft. All authors made significant contributions to the draft and gave final approve for publication.

## Supplementary material

Supplementary Table 1 VENN-gene.fpkm.matrix.annot

Supplementary Table 2 VENN-transcript.fpkm.matrix.annot

Supplementary Table 3 PCA-gene.tpm.matrix.annot

Supplementary Table 4 PCA-transcript.tpm.matrix.annot

Supplementary Table 5 Statistical results of gene expression difference

Supplementary Table 6 Details of gene expression differences in roots

Supplementary Table 7 Details of gene expression differences in leaves

Supplementary Table 8 Details of gene expression differences bZIP-related genes

Supplementary Table 9 Transcription factor prediction results in roots (based on PlantTFDB database)

Supplementary Table 10 Transcription factor prediction results in leaves (based on PlantTFDB database)

Supplementary Table 11 Transcription factor prediction results (based on JASPAR database)

Supplementary Table 12 Statistical results of predicted transcription factor target genes in roots and leaves

Supplementary Table 13 Details of predicted transcription factor target genes in roots and leaves

Supplementary Table 14 Primer sequences for qRT-PCR

Supplementary Figure 1 qRT-PCR verification of genes
