# Supplementary material for: Transcriptome-based analysis of the effects of compound microbial agents on gene expression in wheat roots and leaves under salt stress
Source: Front Plant Sci. 2023 May 10;14:1109077. doi: 10.3389/fpls.2023.1109077 (PMC10206238; doi:10.3389/fpls.2023.1109077)
Supplement: Supplementary Table 8 — Details of gene expression differences bZIP-related genes [file Table_1.docx]

Table S8. Details of gene expression differences bZIP-related genes

| Gene ID | Chromosome location | length | Regulation pattern in root | Regulation pattern in leaves |
| --- | --- | --- | --- | --- |
| TraesCS3B02G411300 | 647819729-647823523 | 3795 | down | up |
| TraesCS3D02G371900 | 485067867-485072396 | 4530 | down | up |
| TraesCS7D02G171300 | 123653486-123658548 | 5063 | down | up |
| TraesCS6D02G312800 | 421879523-421885611 | 6089 | down | up |
| TraesCS1A02G072600 | 56617368-56618763 | 1396 | down | down |
