# Supplementary material for: Transcriptome-based analysis of the effects of compound microbial agents on gene expression in wheat roots and leaves under salt stress
Source: Front Plant Sci. 2023 May 10;14:1109077. doi: 10.3389/fpls.2023.1109077 (PMC10206238; doi:10.3389/fpls.2023.1109077)
Supplement: Supplementary Table 9 — Transcription factor prediction results in roots (based on PlantTFDB database) [file Table_2.docx]

Table S14. Primer sequences for qRT-PCR

| Gene ID | Forward sequence of the primers (5′→3′) | Reverse sequence of the primers (5′→3′) |
| --- | --- | --- |
| *TaACTIN* (AB181991) | CGAAACCTTCAGTTGCCCAGCAAT | ACCATCACCAGAGTCGAGCACAAT |
| *TraesCS3A02G303200* | CTCAACTCCAAGGACTCCAAG | CGCCTGCTCAGTATGCTA |
| *TraesCS4A02G166900* | GAGAAAGAGATTACTGAAGCAG | ATCCTTGTCCAGCTACAAA |
| *TraesCS5B02G214400* | GTGAGGATGAGCCTTTCAAT | GAATGTCTCAGTAGGAGGTTTC |
| *TraesCS5A02G429500* | GTGCAACTGATAGAGTGCTAAA | TCAGGAAGAGGGATGTAGATAAG |
| *TraesCS1B02G372600* | GAGTGGTTCGTAACGGTATAG | GTGCATCAAACCCTTCTATCT |
| *TraesCS7B02G005400* | GGAAATATACTAGGCGCACTC | CGGTAACACCAATCTAACATCTA |
| *TraesCS3B02G318300* | AGGTCTCGCTGCTCGTCTTC | AGGATCTCCATCATTGTTGTTACTT |
| *TraesCS4A02G174100* | GGCCTCGATCTCCTGCTC | TGCTGAGCAGACACAGACAG |
