# Supplementary figures and images for: Transcriptome-based analysis of the effects of compound microbial agents on gene expression in wheat roots and leaves under salt stress
Source: Front Plant Sci. 2023 May 10;14:1109077. doi: 10.3389/fpls.2023.1109077 (PMC10206238; doi:10.3389/fpls.2023.1109077)

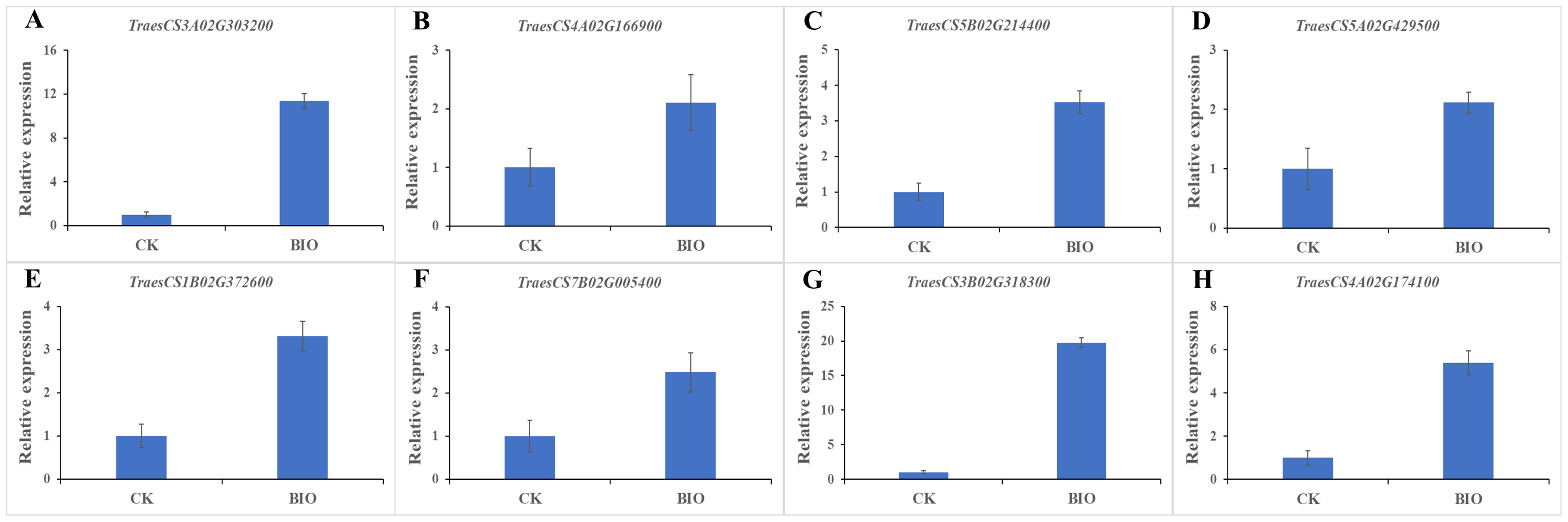

Supplement: Supplementary Figure 1 — qRT-PCR verification of genes [file Image_1.tif]
